# A multimodal domestic service robot interaction system for people with declined abilities to express themselves

**DOI:** 10.1007/s11370-023-00466-6

**Published:** 2023-06-04

**Authors:** Chaolong Qin, Aiguo Song, Linhu Wei, Yu Zhao

**Affiliations:** State Key Laboratory of Bioelectronics, Jiangsu Key Lab of Remote Measurement and Control, School of Instrument Science and Engineering, Southeast University, Nanjing, 210096 China

**Keywords:** Service robot, Older adults, Human–robot interaction, Intention recognition, Multimodal interaction

## Abstract

Driven by the shortage of qualified nurses and the increasing average age of the population, the ambient assisted living style using intelligent service robots and smart home systems has become an excellent choice to free up caregiver time and energy and provide users with a sense of independence. However, users’ unique environments and differences in abilities to express themselves through different interaction modalities make intention recognition and interaction between user and service system very difficult, limiting the use of these new nursing technologies. This paper presents a multimodal domestic service robot interaction system and proposes a multimodal fusion algorithm for intention recognition to deal with these problems. The impacts of short-term and long-term changes were taken into account. Implemented interaction modalities include touch, voice, myoelectricity gesture, visual gesture, and haptics. Users could freely choose one or more modalities through which to express themselves. Virtual games and virtual activities of independent living were designed for pre-training and evaluating users’ abilities to use different interaction modalities in their unique environments. A domestic service robot interaction system was built, on which a set of experiments were carried out to test the system’s stability and intention recognition ability in different scenarios. The experiment results show that the system is stable and effective and can adapt to different scenarios. In addition, the intention recognition rate in the experiments was 93.62%. Older adults could master the system quickly and use it to provide some assistance for their independent living.

## Introduction

As people are living longer, it becomes necessary to provide easier access to assisted living service and care [1]. In addition, the number of patients with motor impairments caused by various diseases such as stroke is increasing [2, 3]. Health emergencies such as coronavirus disease 2019 make the mismatch between the supply and demand of indoor services more prominent [4]. As a result, many resources need to be consumed to take care of these people [2].

To improve their quality of life and provide assistance with activities of daily living (ADL), intelligent service robot system has become a research hotspot [5, 6]. For ordinary users in specific situations, the recognition rate and reliability of a particular modality implemented with the algorithm proposed by the predecessors can meet their set requirements. However, for users who suffer from certain declined abilities caused by physiological and psychosocial changes [1], such as cognitive degradation [7], poor eyesight [8], irregular gesture [9], and ambiguous speech [10, 11], it is difficult to efficiently provide interaction services. Moreover, complex users’ environments, such as different lighting conditions, cluttered backgrounds, and background noise, make the situation more complicated [7, 12].

The interactions among service robots, users, and users’ environments tend to be difficult or even dangerous, which can easily cause unpredictable harm to users and their living environments [13]. As a result, accurate intention recognition and efficient human–robot interaction (HRI) are becoming increasingly important. In order to fully use the service robot system and efficiently provide interaction services, more attention must be paid to users’ physiological and psychosocial changes [1] and the impacts of users’ environments [7, 12].

This paper focuses on addressing the needs of adults with declined abilities to use different modalities who remain able use a screen, understand simple logic, and perform simple manual tasks. Older adults were invited as our primary study population since they are more likely to experience a decline in abilities. To provide some assistance from a robotic-assisted living perspective, we designed a service robot interaction system with an intention recognition method. Based on an investigation of older adults’ experiences and preferences, five independent modalities were used: touch, voice, myoelectricity gesture, visual gesture, and haptics. Virtual reality (VR) technology is employed to allow users to experience the system quickly without needing a long pre-training period to ensure safety. Users’ abilities to use each modality in their unique environments are evaluated without fear of danger.

The main contributions of this paper are as follows:

1. We present a multimodal domestic service robot interaction system to enable people served to express requests for assistance effectively.
2. We propose a method for estimating users’ multimodal inputs and interpreting their intentions.
3. Older adults’ habits and preferences of interactions are analyzed, providing ideas for the future development of service robot interaction systems.

The rest of this paper is organized as follows. Section 3 introduces the overall design of the domestic service robot interaction system, including the system architecture, demand analysis, and a simple description of the service robot. Section 4 proposes a multimodal information fusion method to deal with the multimodal inputs and decision outputs. Section 5 shows the experiments and results. The discussion in Sect. 6 is the further analysis of the experimental results. Finally, some conclusions and future work are given in Sect. 7.

## Related work

*Service Robots and Smart Homes* Many researchers worldwide have studied the design of service robot systems and their interaction methods. A variety of service robots were designed and manufactured with functions covering monitoring, companionship, social interaction, physical support, fetching tasks, etc. [5, 6, 8, 14]. Since 1985, Transitions Research Corporation has been working on the autonomous mobile robot HelpMate, particularly focusing on floor cleaning and hospital transport services [15]. Nurses in the hospital can control it through two main initial interfaces: a screen and keypad on the control console and a voice output system. The robot PR2 developed by Willow Garage can achieve manipulation and self-care tasks functions [16]. Grice et al. developed a web-based augmented reality interface that enables people with profound motor deficits to remotely control it. The Twendy-One robot developed by Waseda University can provide domestic services such as object pick and place, physical support, and kitchen support for older adults and the disabled [17]. During the interaction, voice command recognition and image-based item recognition were used. Pepper, developed by SoftBank Robotics of Japan and Aldebaran Robotics of France, can communicate with people using the touch screen, facial expressions, movements, and voice [18]. Voice interaction and facial expression recognition were also implemented by the service robot Kejia developed by the University of Science and Technology of China [19]. Other notable service robot systems include BHR [20], KONG [21], Care-O-Bot [22], and GiraffPlus [23].

To assist those people in need, technologies such as smart homes can be excellent supplements [8, 24]. For example, a smart house Intelligent Sweet Home at KAIST in Korea based on several robotic agents was developed for the independent living of older adults and people with disabilities [25, 26]. Focusing on home environment, various interfaces such as hand gestures, voice, body movement, and posture have been studied and tested. Another robot-integrated smart home RiSH was presented by Oklahoma State University to provide a physical environment that promotes active aging through the use of a smart home and an assistive robot [27]. The graphical user interface (GUI) was designed for the caregiver and older adults to control the whole system.

*Multimodal Service Robot Interactions* In general, it can be seen that the service robots and smart homes, as well as their HRI technologies, have also made considerable progress. Most of the aforementioned robot systems aimed to serve older adults and people with disabilities, focusing more on assistive functions.

When turning to different application scenarios, these service systems were designed with their own interaction modalities to solve the specific problems. The common HRI modalities mainly include graphical interfaces control [27], voice interaction [28, 29], gesture control [30–32], haptic device control [33, 34], emotion recognition [35, 36], posture and situation recognition [37, 38], etc. Although researchers have noted the importance of HRI and intent recognition for such service systems, they have tried to add external interaction modalities or develop a standard control interface to make the system acceptable and reliable, without dealing with the contradiction and coupling between the interaction results of different modalities.

To deal with these problems, some other researchers have presented their solutions. Multi-round dialogue strategies were designed by Tian et al. [39] to induce users to express their needs, which effectively improved voice interaction and recognition to a certain extent. From another perspective, Werner et al. [9] considered the participant characteristics when studying the interaction of older adults. They evaluated the effects of specific user training on gesture-based HRI between an assistive bathing robot and potential robot users.

To reduce recognition uncertainty, fusing multiple modalities for HRI and intention recognition has become an option that many researchers commonly use [11, 40, 41]. Iba et al. [42] proposed a framework for interactive multimodal robot programming, considering the problem of intention interpretation as a mapping problem from the stream of user inputs, the current state of the system, and the robot sensor data, to the correct robot task. The sequence of multimodal recognition results was mapped to the set of actions using the semantics database. Although the combination of the results from different modalities makes it easier to form a command, when it turns to people with declined abilities to express themselves through different interaction modalities, the command would suffer from the unreliable parts and make the system unstable. Medjahed et al. [43] proposed a domestic healthcare monitoring system and a data fusion approach based on fuzzy logic with rules directed by medical recommendations. Temporary sensor malfunction, user’s environment disturbances, and material limits were considered, enabling the detection of distress events and positions of older adults. However, even without considering the complexity of rule establishment, data that are not concurrent in time, not fixed in the interval, inconsistent in sequence, or irrelevant in extracted features cannot be processed effectively.

When referring to machine learning and neural network in intention recognition, there are also many related excellent works [11, 40]. For example, to recognize human activities, Teng et al. [44] proposed a layer-wise training CNN with local loss to extract features from data collected by different wearable sensors, which can be helpful for situation awareness and user intention recognition. Zhu et al. [45] studied efficient interaction methods in a smart assisted living system for older adults and people with disabilities. Rule-based motion data analysis from the foot and waist sensors is achieved after extracting features using neural networks.

However, most of the neural network training and other data-driven decision-making algorithms suffer from a lack of data sources, especially for older adults or those with disabilities [11]. Their varying degrees of declined abilities lead to more class-imbalanced data recorded from unconstrained conditions. Moreover, the features extracted from the databases are not reliable over time. During the process of long-term use, users will gradually get their system understanding and form a unique expression of preference [40]. There might be many specific interaction patterns and different combinations of modalities for even the same intent. The heterogeneous data with better independence and less correlation from different modalities also make multimodal fusion more difficult. In addition, the various computing times and other resources should always be considered [41].

Some other researchers have tried it from a probabilistic point of view. Liu et al. [46] proposed an architecture based on a weighted fuzzy Dempster–Shafer framework, which can adjust weights associated with inconsistent evidence obtained by different classification approaches, to realize a fusion system (FS) for integrating multimodal information. However, when the number of modalities increases, it remains a challenging and unsolved problem to handle the drastic increase of possible hypotheses/classes and find an appropriate basic probability assignment scheme for allocating support to each possible hypothesis/class. Trick et al. [47] proposed an approach to integrate human advice from multiple modalities into interactive reinforcement learning algorithms. It has been validated to be more robust against misclassifications of the modalities’ individual classifiers. However, it still needs more work to handle learning and preserving human advice over time to enable reusing the given advice during the entire learning process. Whitney et al. [48] defined a multimodal Bayes filter for interpreting a person’s referential expressions to objects. The experiment results showed that it could infer the correct object with high accuracy, but it still required the support of a dataset and has not dealt with the reliability of each modality.

## Overall design of multimodal domestic service robot interaction system

### Architecture

**Fig. 1:**
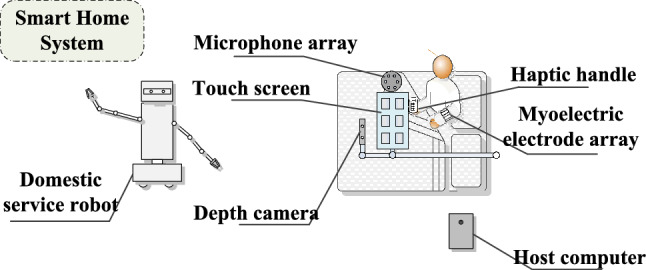
A system application scenario. A depth camera, a touch screen, and a microphone array are installed on a rotatable bedside bracket. A haptic handle [49] and a myoelectric array bracelet are placed at the bedside. A user chooses different modalities to express requirements. Managed by a host computer, the service robot and the smart home system work together to provide some help with independent living, such as fetching tasks and operating home appliances

Our goal is to create a system that is adaptable and free to use to enable effective interactions for people with declined abilities to express themselves through different interaction modalities. Five interaction modalities are employed to satisfy users’ various interaction preferences and expression needs, including: touch, voice, myoelectricity gesture, visual gesture and haptics. A simplified application scenario is shown in Fig. 1. The system recognizes users’ control commands regarding home appliances and service robots through different modalities and responds to the requests for assistance. Aiming to present the multimodal service robot interaction system, the unimodal interaction approach and the modification of the smart home system are presented as system components, which are not the main focus of the work. The smart home system and service robot act as the implementation verification role for interaction responses.

**Fig. 2:**
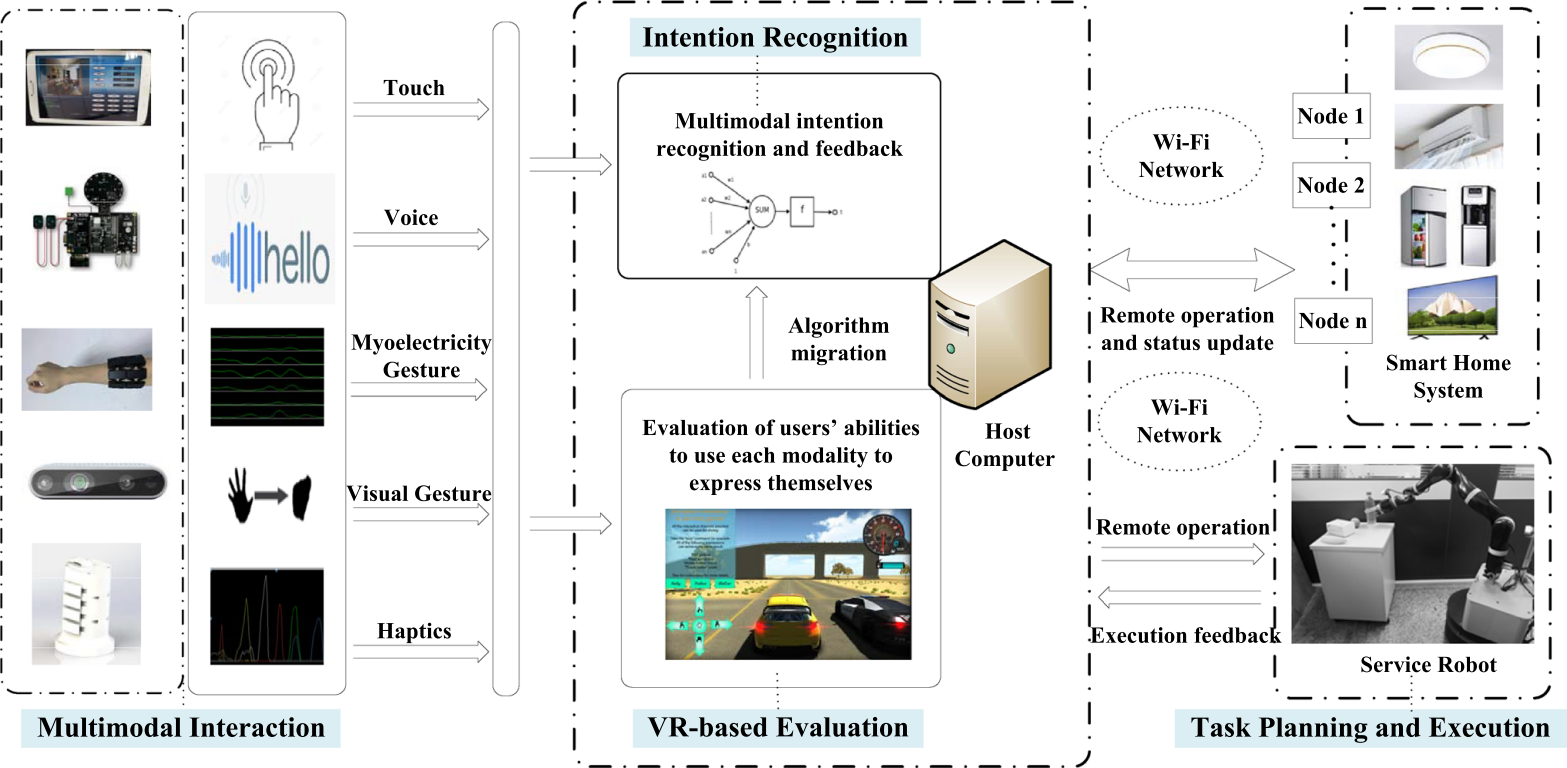
System design. Multimodal interaction is designed for older adults to choose to express themselves. VR-based evaluation is used to complete user pre-training using virtual games and tasks and collect user interaction data of each modality to initialize the fusion recognition algorithm. The algorithm is then migrated for use in practical application environments. A local area network of host computer-service robot-smart home systems is established for interactive control and status feedback

To better collect the information expressed by older adults, interaction devices are mainly installed along the bed with brackets except for a handheld handle and a wearable myoelectric array.

The system can be divided into four modules shown in Fig. 2:

1. *Multimodal Interaction* It includes one or more interaction modalities that users can choose to express themselves simultaneously. The interaction devices used for each modality and their corresponding functions are as follows: the microphone array for sound pickup and noise reduction, the depth cameras for gesture recognition based on color image and depth image, the myoelectric array bracelet for gesture recognition, the haptic handle for haptic feedback control, the touch screen for graphical interface display and touch input. The handle mentioned is developed by our laboratory and can be simplified into a multi-button remote control device with vibration feedback here. The shell of the handle [49] is made of 3D-printed ultraviolet-curable resin with integrated circuitry inside. The pressure of the user’s finger press is detected using Honeywell pressure sensors mounted at the locations of the fingertips when grasping, which can recognize different finger press actions. Further information can be obtained from [51]. Additionally, the touch screen is responsible for displaying the GUI developed based on the Unity3D program [50], which shows the user operation guidance.
2. *VR-based Evaluation* It is designed for data collection and algorithm initialization. Based on VR, some simple interactive games and virtual activities of independent living are designed. The current user is invited to issue commands through each modality separately to assess the ability to use each modality. Finally, the recognition rate of each modality is obtained as the initialization data of the algorithm.
3. *Intention Recognition* In this system, intentions recognized include the selection of task object, the selection of specific operation, and the confirmation of control command. Intent recognition and requirement matching are completed. The recognition algorithm is updated based on the interaction results.
4. *Task Planning and Execution* It responds to users’ requirements, including a Smart Home System and a Service Robot performing tasks and feeding back the execution results. The smart home system and the service robot communicate with the Host Computer through a Wi-Fi network. The appliances in the system are equipped with ESP-01 8266 Wi-Fi modules [51]. A C# program on the Host Computer manages this Smart Home System.

### System workflow

**Fig. 3:**
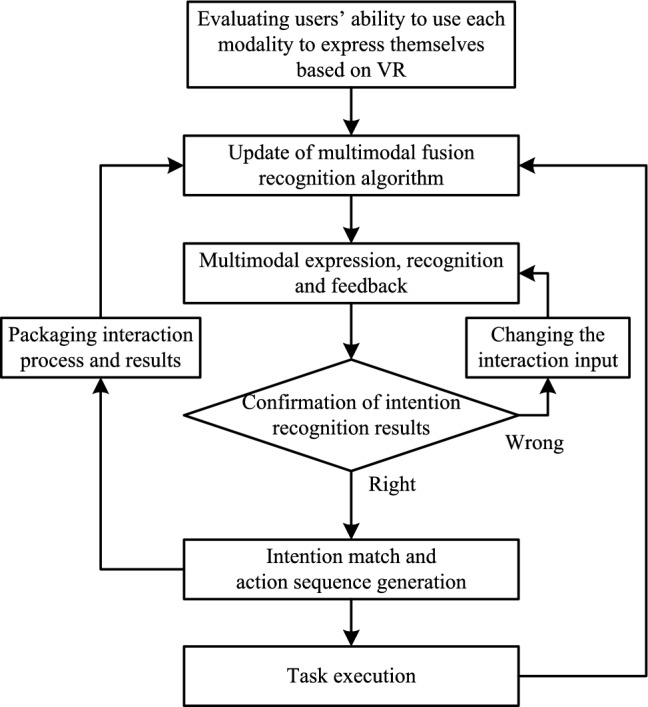
System workflow

Figure 3 shows the workflow of the system.

**Table 1:** Questions for the interview

Number	Questions
1	Do you have difficulties communicating with people in your daily life?
2	What electronic products have you used, and did you have difficulty using them?
3	What are the strengths and weaknesses of these interaction modalities, and how can they be improved?
4	Which modality do you like or accept the most, in order?
5	Are you used to or willing to express yourself in more than one of these modalities simultaneously, and what are they?

First, users can choose whether or not to do a VR-based evaluation depending on the change in the environment and their mastery of the system. Next, a multimodal fusion intention recognition algorithm is initialized. After that, users can start utilizing the system to assist with their requirements. Based on their preferences and abilities to use each modality, users select specific modalities to interact with the service robot system. After confirming the generated command, an action sequence is generated and executed. In addition, the interaction process and results are used to update the multimodal recognition algorithm. Note that it is necessary to provide feedback to the user that the input is received but deemed unreliable. Moreover, the recognition efficiency may be improved by changing the statements or using other modalities with high recognition rates for expression, that is, by changing the interaction input. Therefore, a voice prompt will be given when there is no valid command with high enough reliability in a limited time. The voice prompt will indicate that the system is currently waiting for input and direct the user to change the interaction input.

### Pilot study

Inspired by the human-centered and need-driven design concepts [52, 53], in this paper, older adults, as a group that is more likely to have the declined abilities, are invited as our primary study participants and were involved from the beginning of the system design.

**Table 2:** The details about the modalities selected

Modality	Implementation	Inputs	Outputs	Characteristics	Main requirements
Touch graphical interface (TGI)	GUI developed based on Unity3D [50]	Touch inputs	1. Clicked element	1. Fast and intuitive	1. Electronic product operation ability
			2. Corresponding command number	2. Robust to users’ environments	2. Hand operation ability
				3. Express requirements in steps	3. Ability to see what is on a nearby screen
Voice interaction (VI)	IFLYTEK AIUI cloud platform [54]	Voice signal captured by an array of 6 microphones	1. Formatted semantics	1. Direct, fast and efficient	1. Clear expression
			2. Corresponding command number	2. The more complex the command, the lower the recognition rate	2. Customized recognition of dialects
				3. Susceptible to background noise	3. Background noise cannot cover user expressions
Myoelectric gesture (MG)	An automated data labeling neural network [31]	8-Channel surface electromyography signal	1. Recognized gestures	1. Robust to users’ environments	1. Wear the myoelectric array correctly
			2. Corresponding command number	2. Express requirements in steps with graphical user interface (GUI)	2. Hand movements meet the specifications
				3. Because of the small number of direct gesture commands, commands need to be issued according to GUI	
				4. Ability to see what is on a nearby screen	
Visual gesture (VG)	MediaPipe hands [55]	RGB images	1. Hand skeleton	1. High recognition rate of common gestures	1. No shielding or less shielding of hands
			2.Corresponding command number	2. Express requirements in steps with GUI	2. Hand movement ability
					3. Because of the small number of direct gesture commands, commands need to be issued according to GUI
					4. Ability to see what is on a nearby screen
Haptic handle controller (HHC)	3D printed handle with pressure sensors [49]	Finger presses	1. The pressure values for different positions	Haptic feedback control	1. Hand operation ability
			2. Corresponding command number	Robust to users’ environments	2. Because of the small number of direct gesture commands, commands need to be issued according to GUI
				High recognition rate and fast response speed	3. Ability to see what is on a nearby screen
				Express requirements in steps with GUI	

A pilot study was conducted to find their main concerns and our priorities for technical improvements in system design. A cooperative medical device company was commissioned to invite participants from among their and their partners’ (e.g., nursing homes) customers. Participants should be 60 or older, remain able to use a screen, understand simple logic, and perform simple manual tasks. 16 older adults (9 males, 7 females, between 60 and 82 years old, with an average age of 69.4 and a standard deviation of 6.3) participated in the study. All participants were compensated for their time. After being informed of the purpose of our study, participants were interviewed about their need for expression in daily interactions and their difficulties in using electronic products. The detailed English-translated questions are listed in Table 1.

In addition, we introduced and invited them to experience the interaction modalities through the touch graphical interface, voice interaction, myoelectric gesture, visual gesture, haptic handle controller, facial expression recognition, eye movement control, mouse and keyboard. Our goal was to preliminary validate the feasibility of each modality and interview them about their experience. Therefore, we set tasks as issuing multiple commands that each modality could correctly recognize, such as tapping the touch screen to find the appropriate interface, expressing thirst verbally, wearing a myoelectric bracelet and completing initialization, presenting static/dynamic gestures, holding the handle and pressing different fingers, showing different facial expressions, showing eye movements in different directions, using a mouse to click on icons or keyboard to click on different buttons. Afterwards, they were asked to evaluate the performance of these modalities according to their preferences. During the interviews, participants raised concerns about efficiency, security, and ultimate control. According to their responses, their preferences for the modalities used changed according to specific scenarios that were related to environmental interference with recognition and other personal witnesses. Base on this study, the five modalitiesin Table 2 were selected to compose the multimodal interaction system.

Based on the literature review and pilot user studies, the characteristics of these modalities and the main application requirements for the general implementation we can currently achieve are shown in Table 2. Although the usage requirements for unimodal interactions can be reduced by improving the unimodal interaction algorithm, these usage requirements for each interaction modality will not be eliminated.

In this paper, the recognition rate of these modalities will determine their reliability, on the basis of which the intention recognition algorithm will be initialized and updated. Users can freely choose one or more modalities simultaneously through which to express themselves according to their preferences, and the performance of these modalities in the specific scenario.

### Service robot platform

**Fig. 4:**
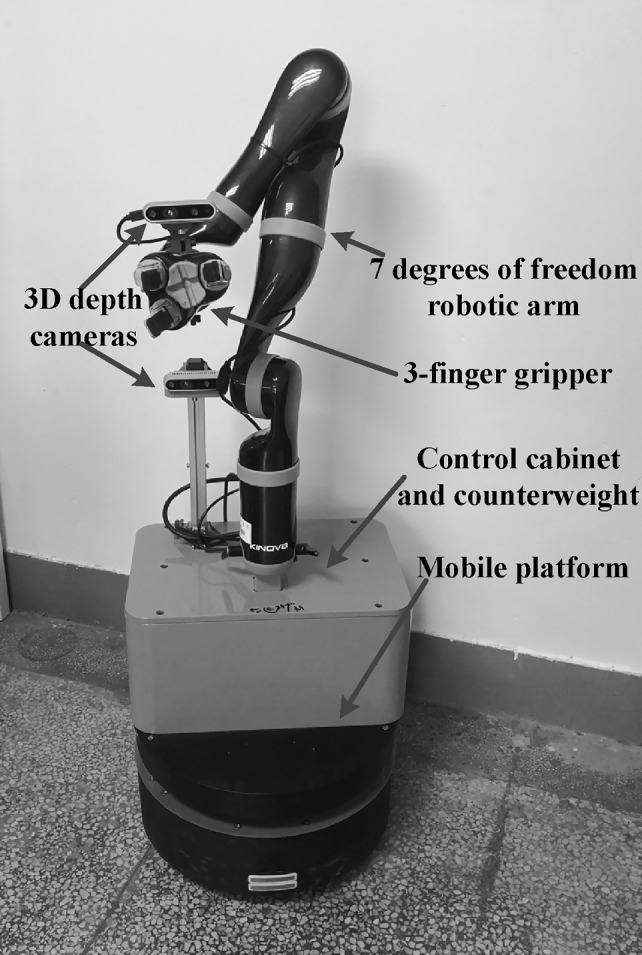
The service robot, providing the platform for testing system performance

Figure 4 shows the designed service robot, which provides the platform for testing system performance.

Considering the actual needs of users with motor impairments, the robot system is implemented under the Robot Operating System (ROS) framework. It is mainly composed of a mobile platform equipped with a robotic arm. The service robot has functions such as navigation, obstacle avoidance, and path planning. The robotic arm uses the Kinova Robotics 7 degrees of freedom JACO2 with a three-finger gripper. Two depth cameras RealSense D435 are connected to the mobile computer in the control cabinet. One is on the platform used for remote item identification and positioning. The other is on the end of the robotic arm used for close grabbing. With the help of these devices, the system can complete fetching tasks, such as fetching a medicine bottle, a water cup, a remote control, or some food.

## Multimodal interaction and intention recognition

It is the primary goal of HRI to recognize the user’s real intention, which is also the prerequisite to meeting the user’s requirements. The intention recognition method based on multimodal information fusion not only needs to solve the synchronization, segmentation, and coupling of the interaction results within a single modality and between different modalities but also needs to make necessary responses to the characteristics of each modality and deal with the changes brought about by the passage of time. Then with the help of the user’s intention coding, the rapid and effective identification and the response of the user’s intention will be achieved.

### VR-based evaluation

To reduce the difficulty of initialization and to obtain the necessary data, instead of evaluating factors such as light intensity and noise level, VR games and virtual activities of independent living are used as a harmless and interesting way to motivate and induce users to complete pre-training. After that,, users can generally perceive their abilities to use the different input modalities, which helps them choose the interaction modalities in the following tasks.

**Fig. 5:**
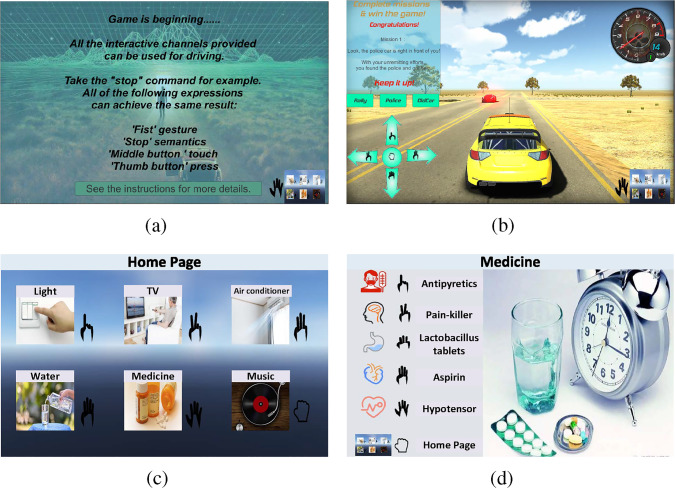
The examples of virtual games and virtual activities of independent living for VR-based evaluation and user pre-training. **a** The game’s initial introduction. **b** The game scene and car motion control corresponding to simple gestures. **c** The main GUI, Home Page, for selecting the task object. **d** The subdivision GUI, Medicine, for selecting specific medicine types. Note that these GUIs were English-translated versions

Figure 5 shows the examples of virtual games and virtual activities of independent living for VR-based evaluation. For example, a game mission to drive to a specified location would appear in the virtual game. The user will be invited to issue commands through a specific modality. Figure 5a shows the game introduction, and Fig. 5b shows the car motion control corresponding to simple gestures. As an example of the virtual activities of independent living is shown in Fig. 5c, d, the user will be asked to express the requirement for a specific drug according to the screen prompt through different modalities.

### Intention recording and coding

With the help of the domestic service robot interaction system, a user can express requirements for assistance and get system responses in time. All requirements are regularized and matched as follows.

- Modality
- Recognition time
- Task object
- Operation performed
- Correctness label

Once the interaction is completed, the fields above will be assigned specific values that will be used to generate the final command. Modality is the source of this recognition result, which is used to mark the reliability. Recognition time is the system time when the recognition is completed. Task object is the object that the current user wants to get or operate, such as water, medicine or the appliances, and the Operation Performed is the specific operation. Correctness label is a flag with different values describing the confirmed correctness of the current result, which we will explain in detail subsequently. For example, when a user says, “It’s dark, turn on the lights, please.” The following result would be achieved: “VI 20:00 light turn on incomplete”.

The correctness of each recognition result cannot be immediately confirmed and applied to the subsequent interaction judgment, which is one of the difficulties in the HRI and intention recognition. Using the progressive relationship between intentions as the benchmark, we confirm the correctness of the recognition result of the previous interaction. The basic “Confirm/Deny” interaction with a high recognition rate is used to reconfirm the final interaction correctness before execution and end a round of interaction.

For example:

*User’s intention recognized 1:Dizziness, need medicine;*

*Robot: What kind of medicine is needed?*

*User’s intention recognized 2: Antipyretics;*

*Robot: I will get you antipyretics right away?*

*User’s intention recognized 3: Good.*

As we can see, there is an intention promotion relationship between the three recognized intentions. The intention 2 is a further refinement of the intention 1. As the interaction process continues, the correctness of intention 1 is confirmed. After the following interactive reconfirmation process, the historical data with the correctness label is updated, making the next round of the HRI process more efficient.

Furthermore, to deal with the continuous interference, if a modality continuously outputs the same command and is confirmed as incorrect twice in a row, the subsequent same recognition result will be considered incorrect.

The correctness label marks the correctness of the recognition result, which takes values in the range {− 1, 0, 1}. The value of the correctness label is 0 for incomplete if the recognition result has not been or cannot be confirmed, 1 for true if the recognition result is output and correct or ignored but incorrect, − 1 for false if the recognition result is output but incorrect or ignored but correct. Only recognition results with a correctness label not equal to 0 are used to evaluate unimodal recognition’s short-term and long-term performance. Details on how to ignore intent recognition results can be found in Sect. 4.4.

Using a GUI, we correspond commands recognized by different modalities to the same intention. Specifically, each modality and the commands they recognized are encoded separately in order. We create a main graphical interface with six subdivision graphical interfaces. As in Fig. 5c, the main graphical interface is used to select the task object, while the subdivision interfaces correspond to different attributes or operations of the task object. Each icon (representing a different task object or operation) can be selected by a label corresponding to a different modality. Figure 5d shows the graphical interface for medicine, and the other subdivision interfaces are similarly constructed. Take “Medicine” in the main scene as an example. It can be selected separately by touching the fifth icon, semantically picking up the medicine, myoelectric gesture No. 5, visual gesture No. 5, and pressing the handle with the little finger, which is numbered 5. This is the first intention output evaluation process: first, each modality outputs recognition results, then the reliability of different results is evaluated, and finally, the final recognized intention is output by evaluating the intention output. Then, suppose the user’s intention is correctly recognized. The GUI will jump to the subdivision interface, as Fig. 5d shows. Subsequently, a similar process of selecting “Medicine” was repeatedly performed on selecting specific medicine types. This is the second time the same intention output evaluation process has been conducted. Finally, the same intention output evaluation process will be conducted the third time in the “Confirm/Deny” process. After the final generated control command is confirmed correct, the robot will be controlled to get the specific medicine. The correctness label will be updated for all generated recognition results in this process, and these results will be used to update the reliability of each modality.

### Correlation of multimodal interaction results

The optional modalities in the system are designed to work independently and produce results in a consistent format, allowing them to be combined freely and integrated at the decision level. The user’s continuous intention expression is discretized in the time domain. The interaction process is segmented to identify which interaction results correspond to the same command. Afterwards, the user’s intentions can be recognized through the association of the interaction results of different modalities.

Maximum duration *T* and the fixed time interval $\varDelta t$ are combined for segmentation. $\varDelta t$ is employed to accommodate the time difference between the recognition results of the selected modalities. *T* is the maximum duration employed for participants to express their requirements. Since we allow participants to correct their expressions, *T* needs to be set long enough to complete two expressions. Note that the final result of unimodality is based on the latest recognition during one segment.

**Fig. 6:**
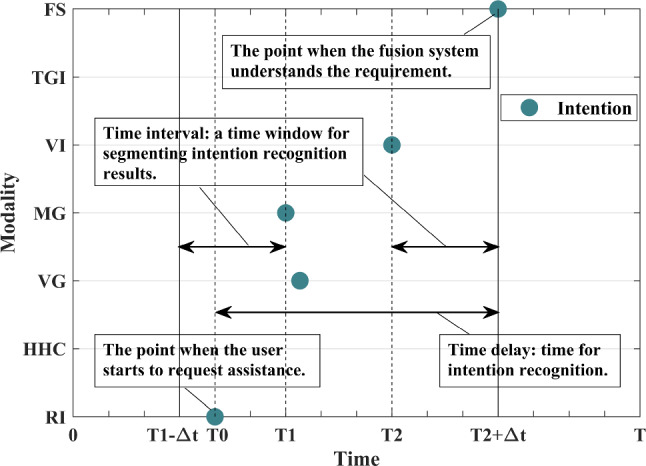
Multimodal output results and time-domain division. The recognition results within $[{{T}_{1}},{{T}_{2}}]$ are preliminarily considered to belong to the same command. RI represents the real intention. FS represents the fusion system

As Fig. 6 shows, the first intention identification result is obtained at ${{T}_{1}}$, the last is obtained at ${{T}_{2}}$, and there is no output result on each modality during the interval $[{{T}_{1}}-\varDelta t,{{T}_{1}})$ and $({{T}_{2}},{{T}_{2}} +\varDelta t]$. The output results in the interval $[{{T}_{1}},{{T}_{2}}]$ are regarded as corresponding to the same demand. The time delay is calculated from the point the user starts expressing the requirement to the point the system understands the requirement, which is less than ${{T}_{2}}-{{T}_{1}}{+2}\varDelta t$.

Suppose there is interference in one or more modalities, and the maximum time interval between the recognized intentions is less than $\varDelta t$. FS will not produce a final result without changing the segmentation method. Therefore, a new segmentation rule is added. In that case, the segmentation starts from the first recognized intention’s recognition time and ends when *T* is reached. If an actual command is issued at this time, the time delay is less than *T*.

### Multimodal fusion

Considering the impact of users’ abilities to use different modalities in their unique environments, we analyze the system performance from the perspective of time change. Users’ essential abilities to use different modalities in their unique environments change slowly. Their physical states, cognitive abilities, and the complexity of the background change little in a short time. However, with the accumulation of experience, people may have varying preferences and levels of understanding when it comes to utilizing the system. In addition, short-term interferences in everyday life scenarios cannot be ignored, such as the incorrect recognition results triggered by noise or misoperation.

Therefore, we process the unimodal recognition results according to Eqs. (1) and (2) and finally obtain the FS output:

1 $$\begin{aligned} {{O}_{f}}=E\circ H\circ {{W}}\circ {{O}_{t}} \end{aligned}$$

Namely:

2 $$\begin{aligned}{} & {} \left[ \begin{matrix} {{o}_{f_{1 1}}} &{}\quad \cdots &{}\quad {{o}_{f_{1 n}}} \\ \vdots &{}\quad \ddots &{}\quad \vdots \\ {{o}_{f_{m 1}}} &{}\quad \cdots &{}\quad {{o}_{f_{m n}}} \\ \end{matrix} \right] {=}M({{e}_{ij}}*{{h}_{ij}}*{{w}_{ij}}*{{o}_{ij}})\nonumber \\{} & {} \quad {=}\left[ \begin{matrix} {{e}_{11}}*{{h}_{11}}*{{w}_{11}}*{{o}_{11}} &{}\quad \cdots &{}\quad {{e}_{1n}}*{{h}_{1n}}*{{w}_{1n}}*{{o}_{1n}} \\ \vdots &{}\quad \ddots &{}\quad \vdots \\ {{e}_{m1}}*{{h}_{m1}}*{{w}_{m1}}*{{o}_{m1}} &{}\quad \cdots &{}\quad {{e}_{mn}}*{{h}_{mn}}*{{w}_{mn}}*{{o}_{mn}} \\ \end{matrix} \right] \nonumber \\ \end{aligned}$$

where $i,j\in Z; i\in (0,m]; j\in (0,n]$. The elements are combined into a matrix, *M*. ${{O}_{t}}$ is the current direct output results of each modality without considering their reliability, ${{O}_{f}}$ is the final output matrix, *E* is the influence matrix proposed for the users’ environments, *H* is the influence matrix proposed for the users’ abilities to express themselves, and *W* is the short-term recognition rate matrix. We encode the modalities from 1 to *m* and the commands recognized by each single modality from 1 to *n*, where *m* and *n* are the number of rows and columns in these matrices. ${{o}_{ij}}$, ${{o}_{f_{i j}}}$,${{e}_{ij}}$,${{w}_{ij}}$ respectively represent their elements, where *i* is the row number, and *j* is the column number. Initially, their values are all set to 1. The element $o_{i j}$ of ${{O}_{t}}$ is a binary variable, taking values in the range {0, 1}, and is determined by the result of each modality interaction. The value of $o_{i j}$ is 1 when the command *j* is recognized by modality *i*. Otherwise, it will be set to 0. The element values of the final output matrix ${{O}_{f}}$ are floating point numbers and populated by the results calculated according to the Hadamard product [56] of the matrix *E*, *H*, *W*, and ${{O}_{t}}$.

Since the reliability is directly reflected by the intention recognition rate, we use the combination of system intention recognition rates in different periods for quantitative analysis to obtain the reliability of different modalities and commands. The correctness label value of ${o}_{ij}$ is defined as ${C}_{o_{i j}}$. The number of recognition results whose correctness label equals 1 can be obtained by $\sum \left( C_{o_{i j}}+\left| C_{o_{i j}}\right| \right) / 2$. The number of recognition results whose correctness label is not equal to 0 can be obtained by $\sum \left| C_{o_{i j}}\right|$. Then the recognition rate of ${o}_{ij}$ can be obtained by $\sum \left[ \left( C_{o_{i j}}+\left| C_{o_{i j}}\right| \right) / \left( 2\left| C_{o_{i j}}\right| \right) \right]$. Note that when calculating recognition rates for different periods, all recognition results for a particular command $o_{i j}$ in that period will be taken into account.

**Fig. 7:**
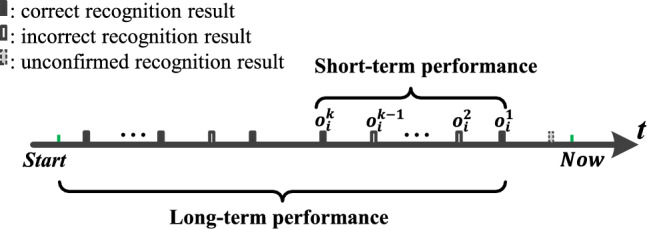
The recognition results of a unimodal interaction. $o_i^k$ is the *k*th recognition result obtained by using modality *i*. The short-term performance is evaluated using the last *k* recognition results whose correctness has been confirmed. Long-term performance is evaluated using all the recognition results whose correctness has been confirmed from system activation to present

Figure 7 shows the recognition results of a unimodal interaction.

The impacts of long-term changes are taken into account by $E\circ H$, which is calculated by the average recognition rate in long-term performance. To simplify the calculation, it is assumed that in the process of the unimodal interaction, the users’ environments and abilities depend only on modality, not the specific command. Therefore, the element $e_{i j} h_{i j}$ of $E\circ H$ can be obtained as follows.

3 $$\begin{aligned} e_{i j} h_{i j}=\sum \frac{C_{o_{i}}+\left| C_{o_{i}}\right| }{ 2\left| C_{o_{i}}\right| } \end{aligned}$$

The impacts of short-term changes are taken into account by a recognition rate matrix *W*, which can be calculated by the average recognition rate in short-term performance. The element $w_{i j}$ of *W* can be obtained by a linear weighted moving average of length *k* as follows.

4 $$\begin{aligned} w_{i j}=\frac{\sum _{l=1}^{l=k}\left[ (k-l+1) \left( {C}_{o_i^l}+\left| {C}_{o_i^l}\right| \right) / 2\right] }{k(k+1) / 2} \end{aligned}$$

When determining the value of *k*, it is important to avoid setting it too large to prevent a slow decline in the reliability of a modality under interference. Instead, the value of *k* can be determined based on the specific interaction performance that is acceptable to participants.

After calculating ${{O}_{f}}$, the sum of the output value of the same command is calculated and sorted. The command represented by the maximum value is used as the recognized intention:

5 $$\begin{aligned} {{o}_\textrm{out}}=\max _{j} \left[ \sum \nolimits _{i=1}^{i=m}{({{o}_{f_{i j}}})}\right] \end{aligned}$$

To deal with interference after a limited amount of misrecognition, a threshold ${o}_\textrm{threshold}$ is set depending on users’ minimum acceptable performance of the system. To compare with ${o}_\textrm{out}$ on the same scale, ${o}_\textrm{threshold}$ is computed as the product of the system’s average recognition rate in long-term performance and the system’s recognition rate in short-term performance obtained using a linearly weighted moving average of the same length *k*. ${o}_\textrm{threshold}$ is considered as a constant output criterion of the system, which is calculated using the system’s recognition results without taking into account the specific modalities used and the commands recognized. When ${{o}_\textrm{out}}<{{o}_\textrm{threshold}}$, ${{o}_\textrm{out}}$ will be ignored as identified interference. In order to achieve an acceptable balance between recognition sensitivity and recognition accuracy, we manually set the value of ${o}_\textrm{threshold}$ in the preliminary experiments.

When the output conditions are met and the user confirms the result, the system will respond in a timely manner. In addition, when the user denies the recognized result, the rest of the commands will be output sequentially based on the above sorted results of the interaction. After the correct user intention is recognized, the parameters of the recognition model will be updated based on the interaction process and the results.

## Experiments and results

In general, we conducted two sets of experiments: the Anti-interference Ability Experiment in the laboratory and the Feasibility Test for Older Adults in a nursing institution. In the laboratory setting, the tasks we verified included normal controls for the home appliances, water and medicine fetching tasks. In the feasibility tests, we are mainly evaluating the performance of the FS in recognizing the intention of older adults. To ensure safety, lift the restrictions of experiments, and improve the experiment’s efficiency, we used voice responses at the beginning and invited older adults to control the service robot and the appliances at the end.

Some hyperparameters determined in this paper were the result of an acceptable balance between recognition sensitivity and recognition accuracy. When participants performed multimodal expressions, the times of the final recognition results for different modalities were close. We finally chose the fixed time interval $\varDelta t = 1.5$ s, which in most cases, can contain all the recognition results and did not significantly affect their experience. Since we allow participants to correct their expressions, *T* was set to 5 s to be long enough to complete two expressions. Note that the final result of unimodality is based on the latest recognition during one segment. We set *k* = 5 for each modality, which is the number of recognized intentions whose correctness has been confirmed in generating two consecutive final commands that are canceled under interference. If it was less than 5, the actual value would be used. Take the medicine fetching task in Sect. 4.2 as an example: 1 Need medicine; 2 Antipyretics; 3 Cancel; 4 Antipyretics; 5 Cancel. Suppose the interference is continuous, then the recognition results will be segmented by 5 s in this paper. The process lasts about 25 s, which will not affect the user’s experience too much. In addition, the minimum acceptable performance was determined based on discussions with users in preliminary trials that the system can only take actions if the average recognition rate in long-term performance was 0.6 and the recognition rate in short-term performance was 0.4. Hence we set our $o_{threshold}=0.6*0.4=0.24$.

### Anti-interference ability experiment

**Fig. 8:**
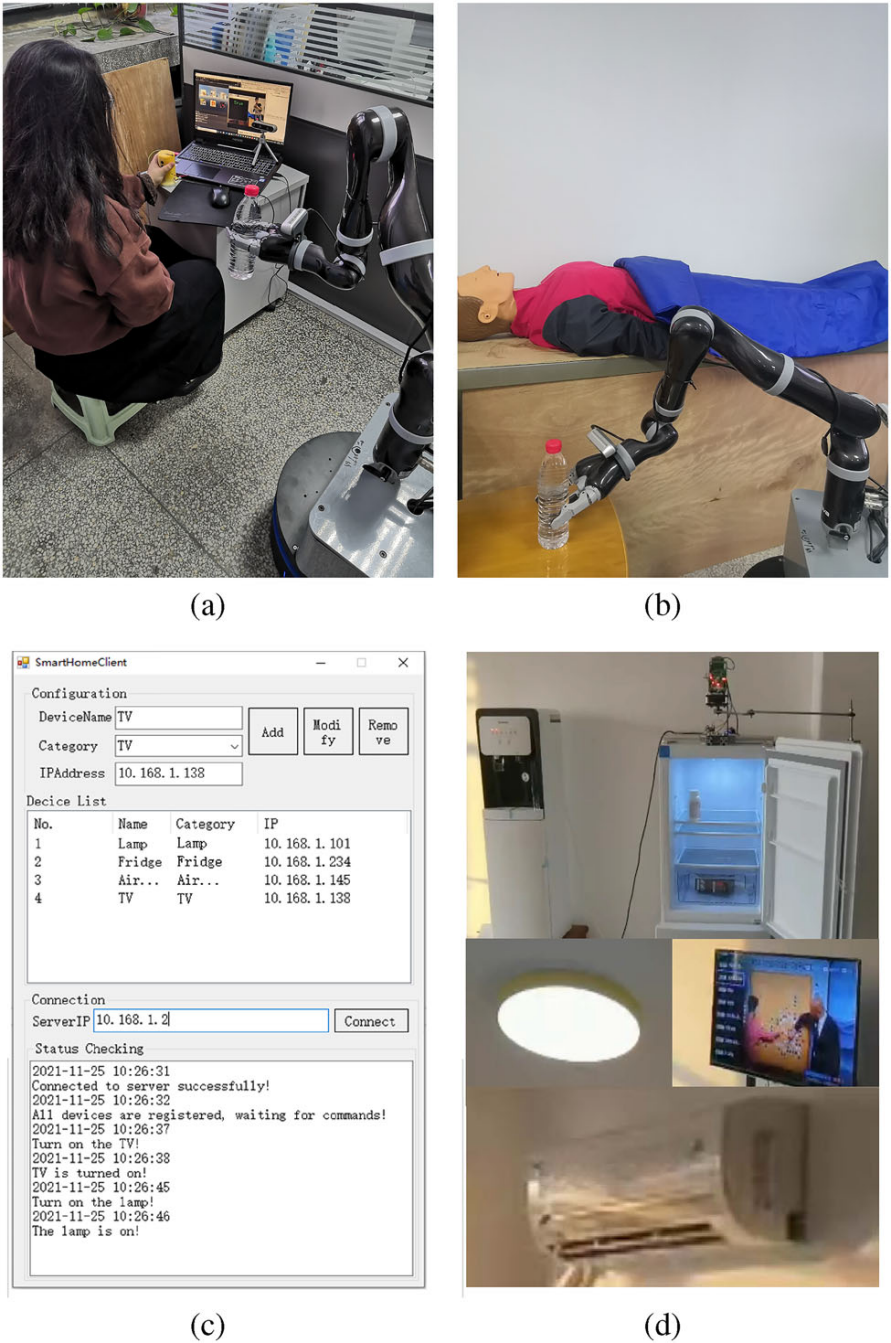
Experimental scenes. **a**, **b** The participant issuing commands using HHC and VI and the robot responding to fetch water. **c** The interactive content of the Smart Home System Manager. **d** The home appliances’ response

The experiment described in this section was conducted by the system developer to demonstrate the system’s immunity to interference. Since the system is fusing at the decision level, interference from different modalities has similar impacts on the performance of the fusion system. Considering the ease of control, HHC was chosen as the modality where interference was present. The handle would be held with a heavy object when additional interference was required. Some commands could be recognized and treated as interference. Note that all the other modalities used during this experiment were controlled to issue the same command to highlight the system performance under interference. Figure 8 shows some experimental scenes.

After the VR evaluation, the recognition rate of each modality achieved is 95% for TGI, 85% for VI, 80% for MG, 90% for VG and 90% for HHC.

**Fig. 9:**
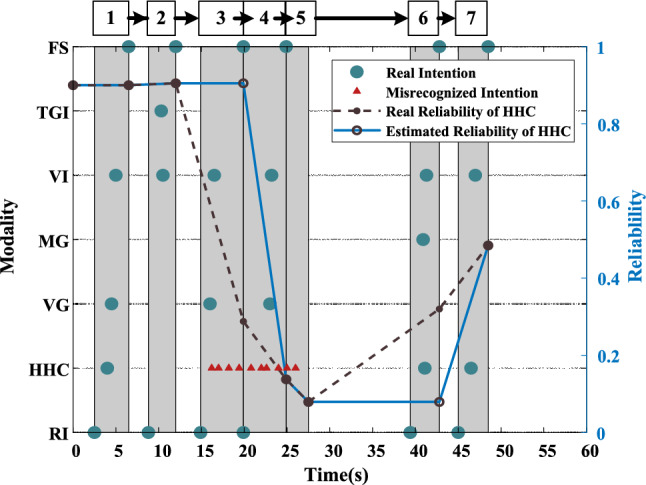
Interaction results and HHC reliability changes. The solid dots represent the intentions. The dashed lines are the real reliability of HHC, which cannot be obtained in time. The solid lines are the estimated reliability of HHC to calculate its output

Figure 9 shows the output recognition results and the reliability change of HHC under the condition of interference addition for the HHC. The red triangles represent the misrecognition results due to noise, and the dashed line represents what may happen when the interference intent cannot be matched. With the help of a context-based reverse update mechanism, which can be found in Sect. 4.2, the reliability of HHC decreased faster when there was interference, and the reliability increased faster when the noise disappeared. The detailed analysis can be found in Sect. 6 Discussion.

**Fig. 10:**
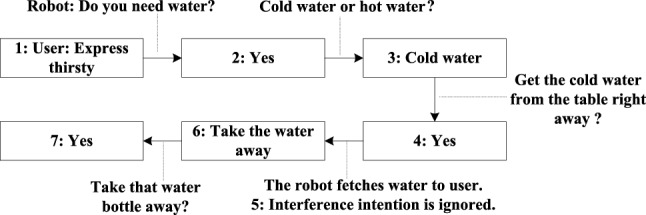
The interaction process corresponding to the experiment shown in Fig. 9

This interaction process can be simplified as Fig. 10 shows. Multiple rounds of interaction were designed in order to present the reliability variation of each modality under different conditions and the context-based update mechanism.

**Table 3:** Each modality output results corresponding to the experiment shown in Fig. 9

	1	2	3	4	5	6	7
FS	2.65	1.81	1.77	1.79	Intent2: 0.14	1.76	0.96
TGI	–	0.95	–	–	–	–	–
VI	0.85	0.86	0.87	0.88		0.88	0.88
MG	–	–	–	–	–	0.80	–
VG	0.90	–	0.90	0.91	–	–	–
HHC	0.90	–	Intent2: 0.90	Intent2: 0.90	Intent2: 0.14	0.08	0.08

The output results of FS are obtained by accumulating the values of the same recognized command by different modalities according to Eq. 5. The output results for each modality are calculated according to Eq. 6

Table 3 shows the output results of each modality and the final system output results during the interaction. Intent2 represents the interference intention and the others represent the real intentions. The results show that in the case of incorrect expression or recognition in a particular modality, the multimodal expression system can deal with the interference and give the user the correct intention options. Furthermore, quick parameter adjustment can lay a solid foundation for the next interaction.

### Feasibility test for older adults

This system aims to serve the particular populations such as bedridden older adults. After the preliminary verification in the laboratory, to test the effectiveness and adaptability of the system, we carried out the feasibility test for older adults.

1. *Apparatus* A microphone array, a depth camera, a myoelectric array, a self-designed haptic handle, and a touch screen were used to form a multimodal HRI module.The detailed information can be found in Fig. 2 and Table 2. The household appliances shown in Fig. 2 and the service robot shown in Fig. 4 were used to form the service system.
2. *Subjects* 10 older adults (4 males, 6 females, between 64 and 78 years old, with an average age of 70.2 and a standard deviation of 4.76) who participated in the pilot study were invited as a convenience sample to conduct the feasibility test. The other 6 older adults could not continue to participate in the experiment for their own reasons. All subjects gave their informed consent before their inclusion in the study. The study was approved by the Ethical Committee of Southeast University.
3. *Experiment design* All older adults participated as individuals. At the beginning of the experiments, each modality and its usage were presented to all participants. A few tasks were performed by participants to ensure that they had mastered the use of each modality.

**Fig. 11:**
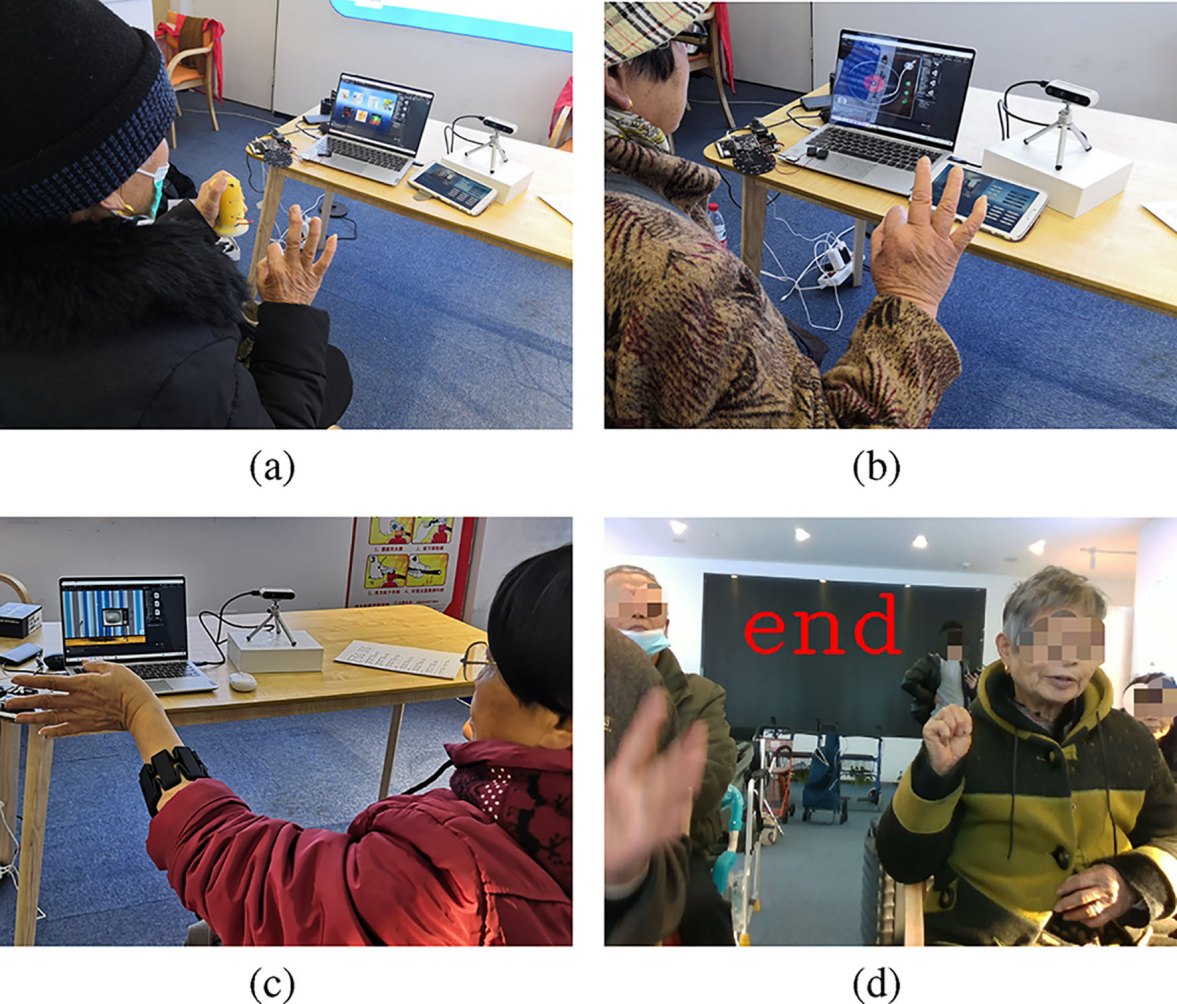
Different practical scenarios. **a** HHC and VG interaction, **b** VI and VG interaction, **c** MG and VG interaction, **d** VI and VG interaction

After the VR evaluation described in Sect. 4.1, six scenarios were set up to allow users to express requirements separately using the five modalities and the FS. Figure 11 shows the different practical scenarios.

Moreover, a balanced Latin square design was used to obtain a set of six modality sequences, including the FS. Each participant was asked to complete six tasks sequentially using different modalities in the designed sequence. For example, there is a modality sequence “Touch Graphical Interface, Voice Interaction, Myoelectric Gesture, Visual Gesture, Haptic Handle Controller [49], Fusion system”. The participant was asked to complete the same task, such as “ask for a cup of hot water”, using each modality respectively in the sequence described above. Then they needed to complete the next task in a different designed modality sequence. Detailed examples of the task completion process can be found in Sects. 4.1, 4.2 and 5.1.

A participant was invited to complete six different tasks using six modality sequences for 36 trials in a round of tests. We did six rounds of tests with each of the 10 participants at different times over 2 days. It took about 10 min for one participant to complete one round. After each round of experiments, participants can fully rest and communicate with the experimenters. Table 4 presents the tasks performed in the a round of feasibility test. The tasks for the other five rounds are outlined but not listed because they are highly similar to the tasks in the table regarding the user interaction process and expression burden.

During the experiments, we did not pursue the optimal users’ environment for recognition, and there are no strict requirements for wearing, background noise, or visual complexity. People can communicate, walk around, and wear face masks or hats. From another perspective, this made the test environment closer to the actual use environment.

According to the design of our interaction interface, each requirement is divided into three parts:

(1) selecting the task object;

(2) selecting the specific operation;

(3) confirming the operation.

As a voice description or a coding combination can provide more information than a simple gesture or a click, the number of interactions is mainly determined by the ability to express themselves using the selected modalities.

Additionally, the user command will be confirmed by multiple rounds of interaction before the service robot performs planned tasks for security reasons. As a result, the interaction effectiveness measured only by interaction numbers is insufficient, especially when compared with other systems. Therefore, we first calculate the correct intention recognition rate to evaluate the intention recognition ability and then evaluate the interaction efficiency and difficulty with the number of interactions required to complete the task.

**Fig. 12:**
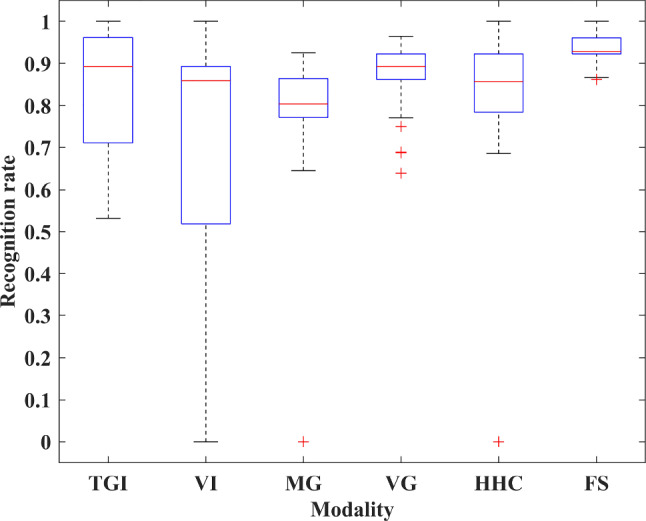
The boxplot of the recognition rates for each modality across all participants in six rounds of feasibility tests. The interpretation of the specific graphic symbols can be found in Sect. 5.2

**Fig. 13:**
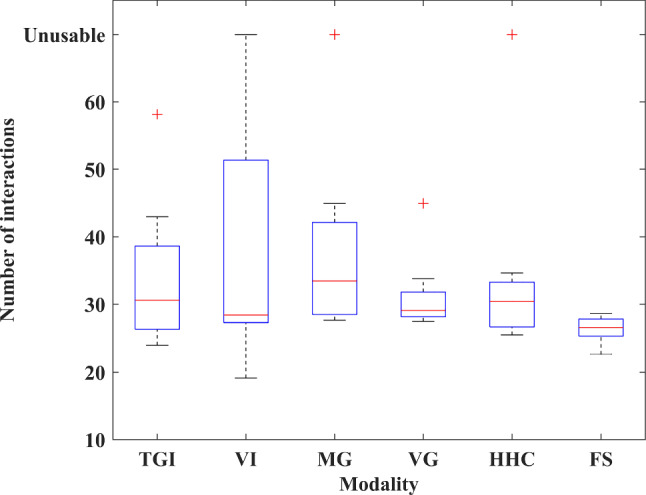
The boxplot of interaction numbers for each modality across all participants in six rounds of feasibility tests. The interpretation of the specific graphic symbols can be found in Sect. 5.2

**Fig. 14:**
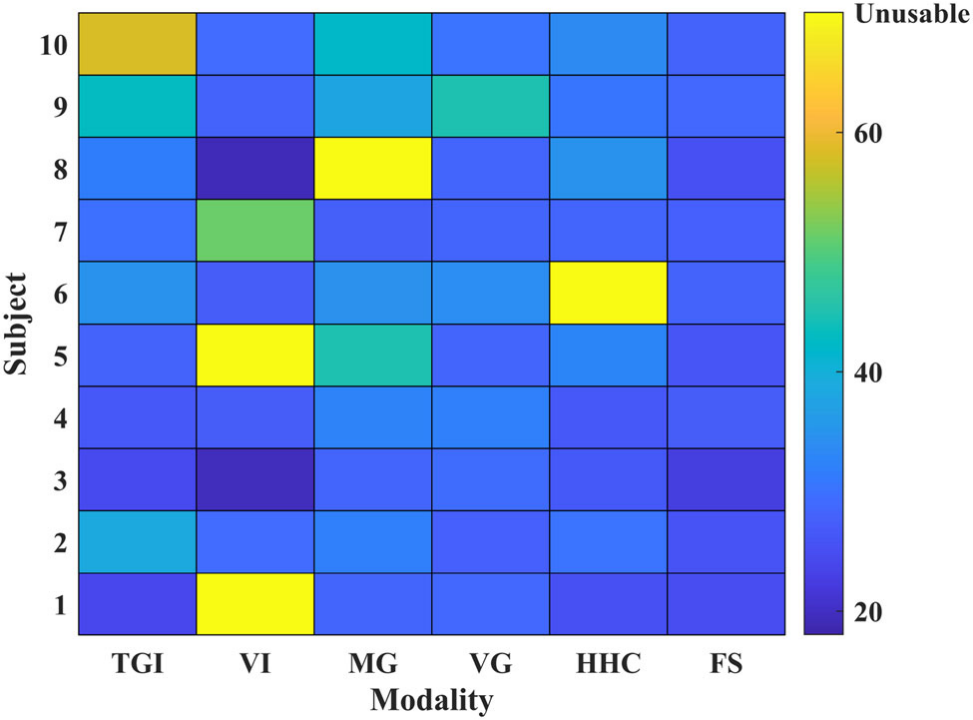
The average number of interactions required for the FS and each modality to work independently to complete one round feasibility test

Table 5 shows the overall recognition results for 10 subjects using each modality. Boxplots are used to show the distribution of recognition rates and interaction numbers. The central red mark indicates the median, and the bottom and top edges of the box indicate the 25th and 75th percentiles, respectively. The vertical whiskers extend from each end of the box to the adjacent values in the data—the most extreme values within 1.5 times the interquartile range from the ends of the box. Outliers are data with values beyond the ends of the whiskers and are plotted individually using the ‘+’ symbol. Note that outliers are flagged, but not discarded. We considered the analysis of outliers as one of the priorities. These outliers revealed differences in people’s ability to use specific modalities. Figure 12 shows the boxplot of the recognition rates for each modality across all participants in six rounds of feasibility tests. With the help of the multimodal fusion algorithm, the set tasks were completed with a total recognition rate of 93.62% and a standard deviation of 3.22%. In general, the recognition rate distribution of the FS is more concentrated and the overall performance is more stable than that of unimodality. Figure 13 shows the boxplot of interaction numbers for each modality across all participants. Figure 14 reflects the interaction efficiency and difficulty with the number of interactions.

**Table 4:** Tasks performed in a round of feasibility test

Number	Task descriptions
1	Get medicine for user
2	Get cold water for user
3	Play some music
4	Turn on the lights
5	Turn on the air conditioner
6	Turn on the TV

**Table 5:** Overall recognition results for 10 subjects using each modality

	TGI	VI	MG	VG	HHC	FS
Means	79.56%	80.19%	81.14%	86.40%	85.71%	93.62%
Ranges	[53.23%, 100%]	[0%, 100%]	[0, 92.59%]	[63.83%, 96.43%]	[0, 100%]	[86.21%, 100%]
Standard deviations	14.57%	35.66%	25.65%	7.19%	27.09%	3.22%

It can be seen that although the number of participants in the experiment is small, due to the lack of personnel selection constraints and specific use environment construction, the difficulty of using each modality for interaction varies from participants. Particularly for voice modalities, the performance difference is the largest. Overall, the FS has the most stable performance among all interaction methods. The detailed analysis can be found in Sect. 6 Discussion.

After the tests, we found that some participants preferred to choose VI and VG for fusion expression. The differences in recognition rates and interaction numbers between VI and FS, VG and FS passed the Shapiro–Wilk normality test ($p>0.05$), respectively, satisfying the conditions for using the paired *t* test. Therefore, the results of these experiments were chosen for analysis using paired *t* test. The paired *t* test results indicated that there were significant differences in the recognition rate between VI and FS, VG and FS, respectively (VI vs. FS: *t* = $-$ 6.032, $p~<0.0001$; VG vs. FS: *t* = $-$ 7.300, $p~<0.0001$). FS was significantly accurate in intention recognition. Moreover, FS requires significantly fewer interactions than VG to complete the given tasks (VG vs. FS: *t* = 7.319, $p~<0.001$). However, there was no significant difference in the interaction numbers between VI and FS (VI vs. FS: *t* = 0.327, $p = 0.746$), which is probably due to the fact that VI can issue commands without relying on the GUI. Significance levels for all analyses were reported at $\alpha$ = 0.05.

**Fig. 15:**
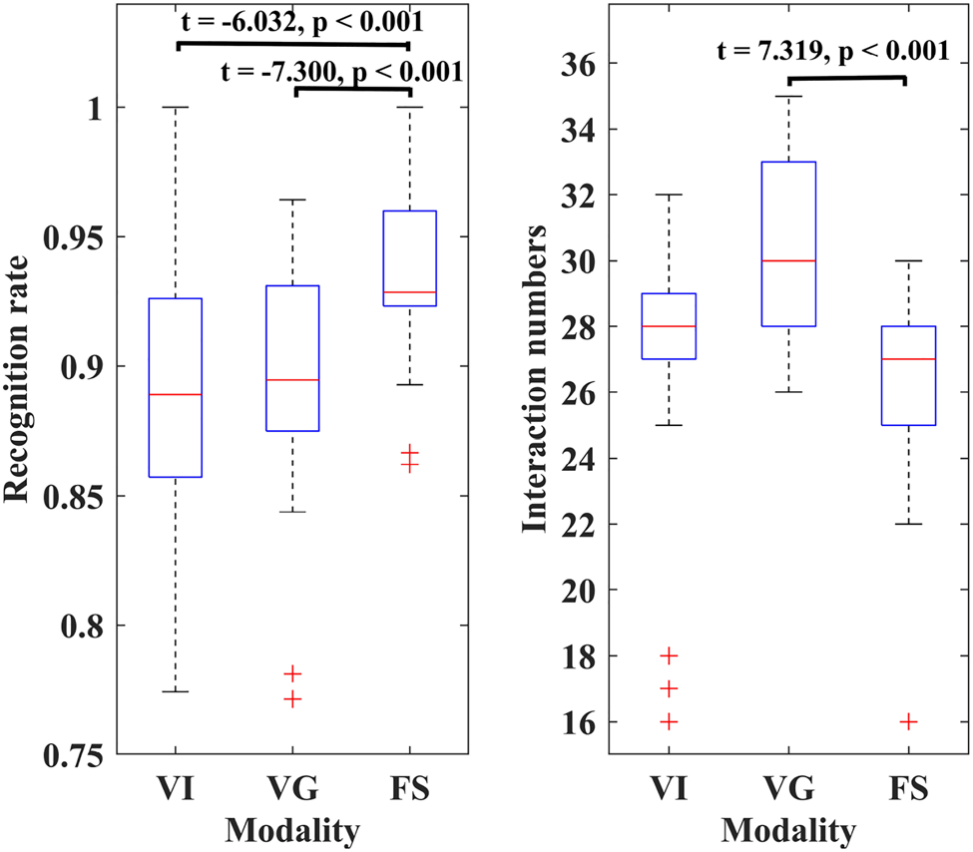
The boxplots of recognition rate and interaction numbers of VI, VG, and FS. The interpretation of the specific graphic symbols can be found in Sect. 5.2

Figure 15 shows the recognition rate distribution of these three modalities. We get the same result, the recognition rate distribution of the FS was more concentrated and the overall performance was better than that of VI and VG.

**Table 6:** Questionnaire proposed at the end of the experiments

Number	Statements
1	I can master how to use the system in a short time
2	Interaction devices do not affect my daily life
3	According to living habits, the system can be used to publish requirements efficiently
4	Commands can be responded to in time
5	The system can recognize my intentions in each round of experiments
6	The system can provide some assistance for my independent living

### User acceptance study

**Fig. 16:**
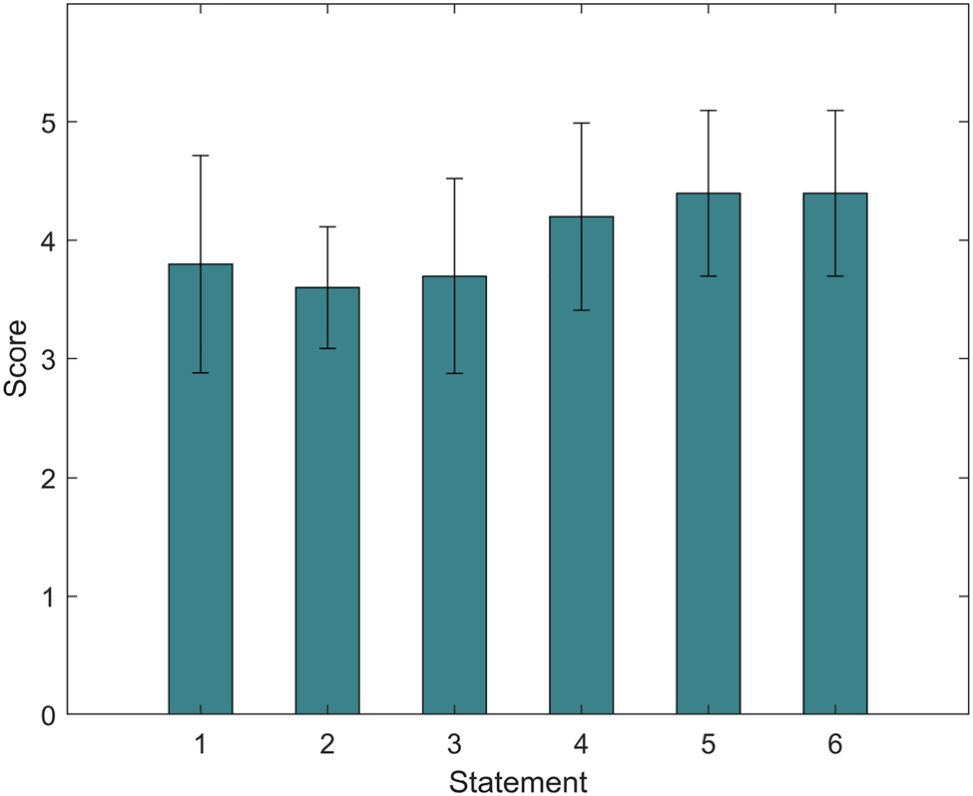
Questionnaire feedback. The bars in the graph represent the means while the vertical error bars represent the standard deviations

Learning from Technology Acceptance Model by Davis [57], a questionnaire designed based on the bipolar Likert five-point scale as shown in Table 6 was used to investigate user experience feedback after the feasibility test for older adults. Result 5 means strongly agree, while 1 means strongly disagree. The specific six statements were evaluated from the learning burden, intrusiveness, interaction efficiency, interaction speed, adaptability, and overall performance. Note that the questionnaire was a translation to English. Figure 16 shows the questionnaire results. It can be seen that the overall performance of the FS is good, especially the performance of statements 5 and 6, which is also the embodiment of our emphasis on system effectiveness and adaptability.

Furthermore, to learn about their perception of the system, we communicated and discussed with them, mainly including their satisfaction with the solution of the problems found in our pilot user study and their suggestions for improving the existing system.

They described the experience with our system as “[intention recognition was] more accurate than the previous [unimodal] experience ”, “[felt] free to express [themselves]”, “easy and convenient [to use]”, “[It would be] better to choose what [they] point at”.

## Discussion

The system obtains a list of possible intentions through one systematic interaction. It sorts the output results according to the reliability of each specific command, which can obtain the user’s intentions at a small cost. From the adjustment process of HHC reliability in Fig. 9, we can see that the reliability of a modality is not constant and is determined by its long-term and short-term performance. Although the HHC has high reliability evaluated by VR, its reliability will be quickly adjusted to reduce the effect of short-term interference. Moreover, this modality will not be permanently deactivated. When the interference disappears, it can be selected for use together with other modalities and subsequently be activated. The high-reliability characteristic brought by the evaluation will rapidly improve its reliability and make it a high-quality modality for the user to express requirements again. However, if the user selects only one modality, continuous interference will cause the problem of unrecognized intention as usual. The difference is that after several interactions, no more wrong commands will be issued, and this interaction modality can only be activated by restarting without the help of other modalities.

From the results of the feasibility test for the older adults, the degradation of users’ abilities to use different modalities, environmental interferences, algorithm limitations, and other factors may limit older adults from using the system for requirements expression. Although the system provides five interaction modalities, most of the older adults have one or more modalities that cannot used freely, such as the VI of users 1 and 5 and the MG of user 8 shown in Fig. 14. Taking their expression preferences into account, there are usually only two or three modalities that fit their expression needs. Moreover, although some modalities have a high recognition rate, users do not necessarily only choose these modalities due to preference and difficulty of use. As shown in Fig. 14, the situation of user 3 and user 8 is the most obvious. Although the number of interactions required to use the voice modality is the least, they do not only use this modality when faced with multimodal selection. Therefore, only relying on a single modality cannot ensure the system’s stable performance. To improve the effectiveness and adaptability of the system, multimodal interaction is indispensable.

It is important to provide secure interactions and services for people with motor impairments in diverse and complex scenarios. Moreover, direct and clear expression of requirements is difficult for our target service groups. Complex and ambiguous expressions can easily cause misrecognition and create a dangerous situation. Low recognition rate may also result in poor user experience and make them refuse to use the service system. Therefore, we divide the interaction process into the selection of task objects, the selection of specific operations, and the confirmation of commands. Although it is not direct enough, the process is self-explanatory and transparent. Users can choose one or more modalities simultaneously through which to express themselves. Although the number of interactions required for the same requirement has increased, it is acceptable according to the user acceptance study and the system performance has been effectively improved. Furthermore, it is necessary to provide feedback to the user that the input is received but deemed unreliable. Guiding the user to perform several trials of different modalities is also necessary for the user and the system to master the user’s current ability to interact with different modalities.

During the feasibility test for older adults, we simulated the real application scenarios as much as possible by not making requirements for the experimental environment and the selection of participants. Ten older adults were invited as a convenience sample to conduct the feasibility test. Although the experiment process is close to the actual application scenario to a certain extent, more long-term feasibility tests under the supervision of experimenters need to be carried on. Moreover, we need a more comprehensive collection of questionnaire results for both the FS and unimodal system separately to make a more explicit comparison and evaluation.

For similar application research on domestic service robot interaction systems, it is necessary to conduct hierarchical confirmations to avoid the possible harm caused by system responses and ensure the safety of participants. To increase the efficiency of HRI, other modalities such as facial expression, habitual action, and body posture are also worth further study. Moreover, the balance between demand mining and respect for users’ autonomous willingness should not be ignored.

## Conclusions

We aim to help people who suffer from certain declined abilities to use different modalities in their unique environments. They can see what is on a nearby screen, understand simple logic, and perform simple manual tasks. After a pilot user study, we design a service robot interaction system and propose an intention recognition method based on the interactions of five modalities: touch, voice, myoelectricity gesture, visual gesture, and haptics, empowering them to perform effective and reliable interactions.

Each modality can be used freely in combination and integrated at the decision level to achieve multimodal fusion to recognize users’ intentions. Under the dual management of the FS and users’ choices out of their preferences, their intentions can be quickly and accurately recognized and responded to by the system.

Furthermore, the system not only considers users’ essential abilities to use different modalities in their unique environments, but also takes temporal impacts into account, such as the short-term interferences and long-time variations. The results of the anti-interference ability experiments and the feasibility test for older adults showed that the multimodal fusion algorithm was effective and stable. The recognition rate was 93.62%. The system was friendly and older adults were willing to accept and use it freely to assist with their independent living.

In the future, more fusion algorithms, as well as the unimodal recognition algorithms, need be studied to improve the performance of our system. Users’ facial expressions and body movements are also worthy of our study to exploit their potential needs. In addition, their emotions should never be ignored. We will apply theoretical frameworks to support our user acceptance study and enhance user experience through timely communication and long-term application. We will strive to help these people in need better as soon as possible.

## Data Availability

The data that support the findings of this study are available from the corresponding author upon reasonable request.
